# Supplementary material for: Conditional chemoconnectomics (cCCTomics) as a strategy for efficient and conditional targeting of chemical transmission
Source: eLife. 2024 Apr 30;12:RP91927. doi: 10.7554/eLife.91927 (PMC11060718; doi:10.7554/eLife.91927)
Supplement: Figure 3—source data 3. [file elife-91927-fig3-data3.zip › Figure 3 source data3 part 1.zip!/Figure 3-Source data 3-part1/1067-9-CRISPResso_Report_c33kf3u3.zip!/CRISPResso_on_1067_11/2b.Nucleotide_percentage_quilt_around_sgRNA_agccaggtgtcgcagacgat.pdf]

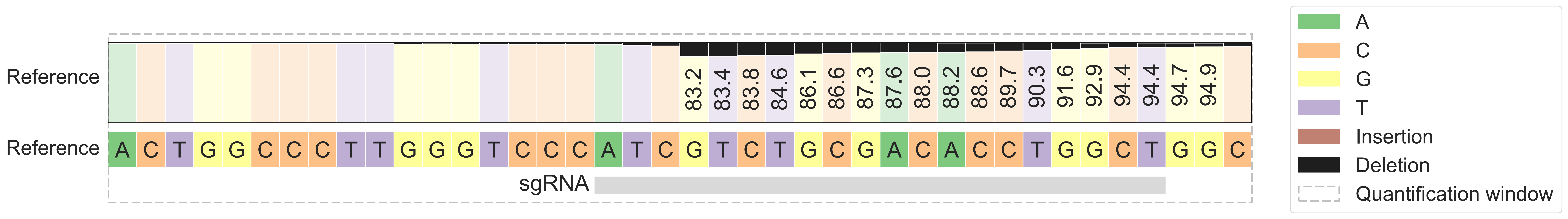
Reference
Reference
A C T G G C C C T T G G G T C C C A T C G T C T G C G A C A C C T G G C T G G C
83.2
83.4
83.8
84.6
86.1
86.6
87.3
87.6
88.0
88.2
88.6
89.7
90.3
91.6
92.9
94.4
94.4
94.7
94.9
sgRNA
A
C
G
T
Insertion
Deletion
Quantification window
